# Diet-Related Disease Prevention in a Rural Australian Setting: Understanding Barriers, Enablers, and the Role of Rural Health Services in Supporting Changes in Local Rural Food Environments

**DOI:** 10.3390/nu15234979

**Published:** 2023-11-30

**Authors:** Nikita Wheaton, Emily Alston, Vincent L. Versace, Michael Field, Anna Wong Shee, Jane Jacobs, Kathryn Backholer, Steven Allender, Melanie Nichols, Cindy Needham, Kristy A. Bolton, Miranda R. Blake, Fletcher Stewart, Evelyn Close, Laura Alston

**Affiliations:** 1 Deakin Rural Health, School of Medicine, Deakin University, Warnambool, VIC 3284, Australia; vincent.versace@deakin.edu.au (V.L.V.); mfield@cah.vic.gov.au (M.F.); anna.wongshee@gh.org.au (A.W.S.); laura.alston@deakin.edu.au (L.A.); 2 Research Unit, Colac Area Health, Colac, VIC 3250, Australia; ealston@cah.vic.gov.au (E.A.); fletch.stewart@deakin.edu.au (F.S.); ejclose@outlook.com (E.C.); 3 Grampians Health, Ballarat, VIC 3350, Australia; 4 Global Centre for Preventive Health and Nutrition (GLOBE), Institute for Health Transformation, Deakin University, Geelong, VIC 3220, Australia; jane.jacobs@deakin.edu.au (J.J.); kathryn.backholer@deakin.edu.au (K.B.); steven.allender@deakin.edu.au (S.A.); melanie.nichols@deakin.edu.au (M.N.); cindy.needham@deakin.edu.au (C.N.); kristy.bolton@deakin.edu.au (K.A.B.); miranda.blake@deakin.edu.au (M.R.B.); 5 Institute for Physical Activity and Nutrition, Deakin University, Geelong, VIC 3220, Australia

**Keywords:** rural health, prevention, food environments, retail interventions, nutrition, Australia, Victoria

## Abstract

Bold and comprehensive action is needed to prevent diet-related diseases in rural areas, which includes improving food environments to enable healthier dietary practices. Rural health services are integral to the health of rural populations, yet their role in community disease prevention is not swell understood. This study sought to understand health service, local government, and food outlet stakeholders’ perspectives on (1) the drivers of unhealthy retail environments in a rural setting; (2) the role of rural health services in supporting changes in local food environments; and to (3) identify characteristics of potential interventions. Two Group Model Building workshops were held with health service and local government leaders (n = 9), and interviews were conducted with local food outlet participants (n = 13). Key themes included ‘enablers to healthier food environments’, ‘barriers to healthier food environments’, ‘Rural health services are a leading broker of knowledge for healthy food environments’, and ‘characteristics of desirable healthy food environment interventions.’. Rural health services can play a key role in addressing the current barriers to healthy food environments in rural areas. Effective promotion of healthier diets in rural populations will require consideration of key stakeholder perspectives and the development of further evidence on the role that rural health services can play in improving the healthiness of food environments.

## 1. Introduction

Sub-optimal diets (i.e., overconsumption of energy-dense nutrient-poor food and inadequate fruit and vegetable intake) are a key driver of inequalities in preventable disease burden in rural communities compared to metropolitan counterparts [1]. Modelling studies have demonstrated that up to 38% of the gap in cardiovascular disease mortality rates between metropolitan and rural Australia is related to modifiable risk factors such as diet and obesity [2]. Studies have shown access to and the promotion of healthy food, such as higher-nutrient and less-processed food, is more limited in rural food retail settings when compared to metropolitan settings [3,4]. Despite this, there has been limited action in rural and non-urban food environments globally, and little is known about how best to improve the healthiness of food environments in rural areas. Further, critical funding gaps in Australia have led to a lack of research and evidence to inform future initiatives to improve the nutrition of populations living outside of major cities [5]. 

To date, there are no studies on rural health service-supported interventions to improve community rural food environments, either in Australia or globally. In this paper, rural Australian communities are those defined as Modified Monash Model (MMM) areas 2–5, i.e., regional centres (population >50,000), large rural towns (population 15,000–50,000), medium rural towns (5000–15,000), and small rural towns (<5000) [6]. A qualitative Australian study involving public health policy makers found that the lack of published scientific evidence from outside major metropolitan areas limits the application of evidence across rural communities. Regional and Rural Health Services (RRHSs) in Australia have been acknowledged to play a pivotal leadership role in improving health and influencing the wider community by promoting and supporting health-enabling environments [7,8]. However, RRHSs are often smaller than their metropolitan counterparts and experience specific challenges, such as resourcing and workforce retention [8,9]. Recently, research has conceptualised the leadership role of health services in obesity prevention; however, empirical data on the topic is lacking in Australia [10]. Theoretically, rural health services play a larger role in the economy of their catchment, beyond the provision of healthcare [11]. The high social capital of RRHSs, which typically employ many community members enabling a wide reach across the community, places these organisations in an ideal position to drive health promotion. This could also extend to supporting local rural communities to drive healthy food environment change [11]. Qualitative exploration of multi-stakeholder views on the roles of rural health services does not exist in the Australian context, or more broadly. 

There are several key perspectives to consider in addressing the healthiness of rural food retail environments, with the views of independent community food outlet owners, health services, and the local government being central in this context. Understanding how RRHS, local governments, and food outlet owners can work together in the rural context to improve food retail environments has not been comprehensively explored in the literature, despite intersects of government policy across the three stakeholders [12,13,14]. RRHS and local governments work closely to deliver community health, promotion, and education services, through funding from state governments in Australia, such as in Victoria [12]. In Victoria, for example, one mechanism for policy leverage is the means by which local governments could regulate food retail environments as part of their statutory requirement to register them under the Food Act [13], legislative requirements to deliver public health and wellbeing plans [14], and the work they do jointly on health promotion projects with rural health services [12]. Understanding the potential for these organisations to work together to address the healthiness of rural food environments remains a critical knowledge gap. 

Other international studies seeking to understand the barriers to improving the healthiness of food retail environments have cited a lack of resources [15,16,17] and a fear that consumers may have negative reactions to changes to food outlet offerings [15,16,17]. In addition, barriers to storing healthy food [16,17] and restrictions around existing contractual agreements with suppliers both inhibit progress in improving the healthiness of retail environments. For example, a study in rural British Columbia, Canada, sought food outlet perspectives on potential interventions for improving the healthiness of grocery stores and also described similar challenges to improving the healthiness of their outlets, including issues with storing perishable food and barriers with supplier contracts [17]. In the rural context, featuring local produce and focusing on supporting the local community has been perceived to be a key enabler for improving the healthiness of rural food environments [17]. These studies have not included rural health service stakeholders in either understanding the barriers or solutions to improving local food retail environments. 

The aim of this study was to understand health service, local government, and food outlet stakeholders’ perspectives on (1) the drivers of unhealthy retail environments in a rural setting, (2) the role of rural health services in supporting changes in local food environments, and (3) to identify potential characteristics of interventions.

## 2. Materials and Methods

### 2.1. Setting

This study was set in a rural Victorian Local Government Area (LGA), which had a population of just over 22,000 people in 2021, a median age of 45 years (compared with the state median of 38) [18], and covered 3438 km^2^ and covers areas classified as both ‘small rural towns (MMM5)’ and ‘medium rural towns (MMM4)’ [6]. The largest town in the LGA is situated approximately 70 km to the nearest Metropolitan area (MMM1). In 2020, this LGA had an obesity rate of 27.5%, which is above the state average of 20.9% [19].

### 2.2. Design

This study used qualitative methods from community-based system dynamics [20,21] to map factors that influence healthy food environments from the perspective of health service leaders, local government leaders, and local food outlet owners/operators (using Group Model Building (GMB)) [20]. We also utilised one-on-one qualitative interviews in order to gather data from retailers at a time that was convenient to them. The participatory GMB process allows all participants to contribute and promotes ownership of the actions presented in the Causal Loop Diagram (CLD). The systems methodology allows all community participants to appreciate the complexity of food environments and enables them to visualise and design actions across multiple parts of the complex system in their community [20,22]. The current obesity prevention literature [23] and guidelines from the World Health Organization [24] recommend systems-based approaches in the design and implementation of interventions for sustainable obesity prevention. Community engagement and the systems dynamics methodology have been used in obesity prevention to map community perspectives on various community issues and to catalyse action in multiple regions [25,26,27,28]. Ethics approval for this study was obtained from Deakin University (HEAG_H 107_2019) and Barwon Health (21/107).

### 2.3. Theoretical Framework

This study is underpinned by both social constructionist [29] and systems dynamics theories [30], whereby the socially constructed complexity of interacting components from different stakeholders is acknowledged and understanding is sought.

### 2.4. Participants and Recruitment

Participants, at the time of recruitment, were adults over the age of 18 years who were either health service leaders, local government leaders, or food outlet owners, operators, or managers in the community. 

Health service and local government leaders were recruited via email addresses that were available to the researchers through existing networks or publicly available through the health service’s website. A snowball sampling approach was utilised, and leaders were asked to suggest other potential participants who may be able to contribute to the study. Health service and local government leaders were operating at health promotion, management, executive, or board levels. A letter of invitation was emailed with the study outline, the plain language statement, and a consent form. Stakeholders returned the consent form to the researcher to confirm their participation.

Food outlet participant eligibility included being an owner, operator, or manager as these positions hold authority and have the ability to drive change in their food outlets. Chain outlets where the manager did not have the authority to implement changes, (e.g., fast-food franchise outlets) were not eligible to participate. A list of food outlets in the local government area was collated through publicly available listings (e.g., an online telephone directory), and the research team used these listings to conduct in-person recruitment at the beginning of the project. This involved LA and NW visiting the food outlets and discussing the project with them. Each identified stakeholder was provided with a hard copy outline of the study, a plain language statement, a consent form, and the principal researcher’s contact details. During the discussions, there was an opportunity to ask questions about participation. Stakeholders returned a signed consent form to the researcher to confirm their participation.

### 2.5. Data Collection

Data were collected online via two GMB online workshops with local government, research, and health service staff (n = 9 in each session). A further n = 13 face-to-face, semi-structured interviews were completed with food outlet participants at their workplace. Food outlet participants could not attend the GMB online workshops due to time constraints and varying availability; hence, semi-structured interviews were completed with the food outlet participants at a time convenient to them. This was also utilised to ensure the power balance resulting from the Food Act and the powers of local government over food outlet owners were minimized [13]. Online delivery of GMB group workshops was developed during COVID-19 [28]. The online workshops and face-to-face interviews explored what factors are thought to be driving the unhealthy food retail environment, the potential role of the RRHS, and what actions could be undertaken to address this complex issue.

#### 2.5.1. Group Model Building

The workshop techniques were informed by community-based system dynamics [21] and guided scripts, as well as a previous study on the food retail environment in the same local government area [3]. Previous food environment analysis in this region provided data to inform the need to understand ways to improve the healthiness of the local food retail outlets [3]. In the first workshop, local government and health service staff were asked “What affects the food retail environment in <insert name of LGA>?” and “What is the role of the rural health service in improving the food retail environment?” This information was then collated using Systems Thinking for Community Knowledge Exchange (STICKE) software [31] to create a visual representation (Causal Loop Diagram or CLD) of the group’s shared understanding of the problem. The CLD with local government and health service staff was created in real time, and the session’s audio was recorded and professionally transcribed. The transcript was used by the researchers (LA, NW) to verify and refine the CLD. In the second workshop, the CLD was verified by the group, and participants generated and prioritised actions that might be taken to improve the healthiness of the food environment within their community. Such intervention ideas were discussed with food outlet participants in the semi-structured interviews.

#### 2.5.2. Semi-Structured Interviews with Food Outlet Participants

The discussion guide for semi-structured interviews (see Appendix A) with food outlet participants was based on the GMB scripts to allow thematic analysis of both the GMB and interview transcripts. Participants were asked the same series of questions, as well as reviewing and consolidating the CLD developed in GMB 1 and 2, and the participants discussed intervention ideas. Interviews were recorded using an audio device and then transcribed verbatim.

### 2.6. Data Analysis

Interviews were conducted until data saturation was reached [29]. Following transcription, the interview and GMB workshop transcripts were analysed jointly according to the five-stage framework for reflexive thematic analysis, developed by Ritchie and Spencer [32], which includes familiarization, identifying coding framework, indexing, charting, mapping, and interpretation. An initial coding framework was developed by LA and NW using two transcripts generated from the GMB sessions (deductively underpinned by the aims of this study and similar research) [33]. Two further members of the research team (FS, EC) used this framework to code the remaining transcripts using Microsoft Excel. Two members of the research team (LA, NW) used this framework to code all interview transcripts deductively. This dual-coding approach allowed for the discussion of codes and a richer interpretation of data, leading to consensus on the key themes. Following the coding process, data were analysed to identify patterns in interview responses.

Themes were used to build a joint CLD and were cross-checked with the results of the two online workshops and thirteen interviews (NW, LA). Differences were discussed (NW, LA) and the CLD was edited to best capture the overall perspective of the community. Anonymous quotes are included from GMB and food outlet participants to provide further insight into the themes.

## 3. Results

### 3.1. Participants

Six health service and three local government leaders participated in both the online GMB workshops. Thirteen participants from food outlets completed the semi-structured interviews.

### 3.2. Summary of Overall Themes

Four key themes were identified across the three participant groups, including, ‘enablers to healthier food environments’, ‘barriers to healthier food environments’, ‘Rural health services are a leading broker of knowledge for healthy food environments’, and ‘characteristics of desirable healthy food environment interventions’ outlined in Table 1 (also see Supplementary Table S1).

### 3.3. Local Government and Health Service Causal Loop Diagram

**Figure 1:**
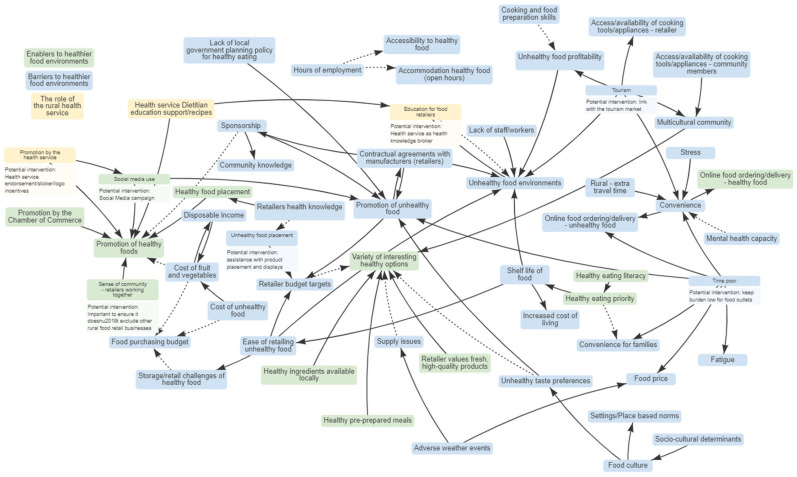
Causal Loop Diagram describing factors that influence the local rural food environment. (full line—as one variable increases the other increases; dotted lines—as one variable increases, the other decreases).

Participants described the system influencing the local rural food environment. Their responses are illustrated in the CLD (Figure 1) as three colour-coded themes: (1) enablers to healthier food environments (green); (2) barriers to healthier food environments (blue); and (3) the role of the rural health service as a leader and knowledge broker (yellow). During GMB2 and the interviews, characteristics of desirable interventions as suggested by the participants were added to the related variables. These themes and intervention suggestions also arose from the one-on-one interviews with food outlet participants and were added to the CLD. The CLD enables community participants to visualise and understand the complexity of determinants influencing the food environment. This allows participants to start to think about ways in which interventions and actions may be able to intervene within the food environment.

#### 3.3.1. Enablers to Healthier Food Environments

Participants (GMB and interviews) were willing to move towards healthier retail environments, despite their perception that the current system does not support healthy food environments. Participants expressed that there were multiple enablers to healthy eating in the rural food retail environment and acknowledged that there is evidence demonstrating that placing healthy food so it is visible to the customers plays a role in the purchasing behaviours of customers. 

Locally available healthy options and ingredients were important enablers for promoting healthier food environments from the food outlet participants’ perspectives. Social media was also identified as an enabler for some food outlet participants, highlighting an easy way to advertise healthy meals to customers.

‘*I haven’t actually found anything that has stopped us from doing what we wanna do. We’ve been able to source local produce of numerous suppliers, which are all homemade or locally made. And then we also make them ourselves. So produce has been quite easy to find*’

Interview participant #12 (Food outlet)

One food outlet participant summed up the willingness to provide healthy food to the local community as:

‘*…what people are offered is what they’re gonna get. So, that’s why here, we try to make it a—only fresh produce, healthy produce…..I think that’s what [Local Government Area] wants and needs. And there are a couple of places around town now that offer it. So, if we can keep going with that, I think we’re on the right track*’

Interview participant #12 (Food outlet)

There was recognition that some customers prefer to have healthy options when eating out. Some food outlet participants also noted that unless there were healthy options available, they may lose business, and this was underpinned by the perception that the local community sought healthy options from retailers.

‘*Well, I think obviously, as a business, you lose business if you’re not gonna meet demands and meet needs, but in terms of affecting business, like you lose business, like after a swim or a gym or something like that, they don’t necessarily want a chocolate or anything like that. So, if you don’t have anything healthy on offer, they’re not gonna buy from you*’

Interview participant #11 (Food outlet)

#### 3.3.2. Barriers to Healthier Food Environments

Despite agreement on the enablers of healthier food environments, food outlet participants, local government, and health service leaders identified complex barriers that the rural community faces in accessing healthy food and promoting healthy food environments. These included existing contractual agreements with manufacturers, local government planning policy, community mental health stressors, a preference for convenience, and poor access to high-quality healthy food.

A local government leader acknowledged that contracts with food and drink suppliers are a major issue for improving the healthiness of food environments across the region. They acknowledged that this was difficult even in the health service setting, let alone how hard it would be for private retailers to negotiate.

‘*…when we were doing the choose water campaign <health service name> were looking at putting water in all their vending machines, but the contract was with <drink name> would say that they needed to have certain products for those machines. Because that was part of the deal, of having the machine*’

GMB1 Participant #7 (Local Government)

Another participant acknowledged current government policy as a barrier to a healthier rural food environment. Participants highlighted that there was no control over the number of types of food outlets that were able to be set up in the township, as well as no limits on marketing.

‘*To my knowledge, there’s nothing in the planning scheme that would prevent, say, a fast-food outlet from establishing itself anywhere in the Shire as long as the zoning is correct. Same with, say, their signage or their marketing. A healthy outlet has to abide by the same rules as an unhealthy outlet. So there’s nothing in the planning scheme that local government can enact to change that*’

GMB2 Participant #3 (Local Government)

When asked about barriers to healthier food environments, the participants acknowledged the dominance and convenience of unhealthy retailers, for example, fast-food franchise delivery services.

‘*Maccas [McDonalds] and KFC [Kentucky Fried Chicken] do home deliveries now*’

GMB1 Participant #7 (Local Government)

When questioned about what affects the local food environment, cost was one of the most important barriers for food outlet participants in working towards healthier food environments. This was further impacted by the shelf life of healthy foods; healthy products tend to have a shorter shelf life and a greater likelihood of increased food wastage and lost revenue. Although acknowledged as a barrier, some food outlet participants felt it was important to spend money on fresh produce.

‘*And in terms of shelf life, we tend to throw a lot of our fresh products. Cost would be a massive thing. We currently sell sandwiches and they’re seven dollars a pop. We’re throwing them out every two days ‘cause it’s just not what people wanna spend that amount of money on*’

Interview participant #11 (Food outlet)

Several participants referred to skills and knowledge as a key barrier to consumer preferences of what constitutes a healthy food environment, as well as food outlet owners and staff’s understanding of healthy foods versus dietary requirements, with many food outlet participants assuming that vegan and gluten-free products were healthy options.

‘*…we do have things like—we do vegan options which I guess ultimately vegan is quite healthy and we have cold options there. So just confirming what are the healthy options in our business. I mean, we obviously think that vegan’s healthy. We’ve got cheese and salad rolls that are healthy, so we do a vegan sandwich*’

Interview participant #10 (Food outlet)

#### 3.3.3. Rural Health Services Are a Leading Broker of Knowledge for Healthy Food Environments

All participants acknowledged that the local rural health service could play a role in assisting local food outlets as the health knowledge broker in addressing barriers and also in augmenting facilitators of healthy retail food environments. They acknowledged that food outlet owners want to make their food environment healthier but do not necessarily know what is healthy.

‘*There was a lot of like (we) promote like vegan and gluten free… Without understanding that that was promoting a healthy option when really that’s just catering to different dietary requirements*’

GMB1 Participant #1 (Health Service)

They also acknowledged the health service as a vector for knowledge on healthy food availability and promotion and that community-facing dietetics/health promotion staff members could help food outlet participants make decisions about how to make their menus healthier.

‘*Yeah, that [having a dietitian], would be beneficial like them to sort of have a look at the menu and go, “Well, yes, this is fully healthy,” or, “This is like half healthy. But if we did this, it would be fully healthy.*’

Interview participant #13 (Food outlet)

Food outlet participants acknowledged the role that the health service could play in which they support healthy changes made through reward systems and assessment. An example of the health service providing promotional stickers that are endorsed was mentioned by retailers, and the health service could promote businesses with healthier food environments to patients of the health service.

‘*…some sort of sticker to stick on my label saying this is a healthy choice option*’

Interview participant #10 (Food outlet)

As the main organisation providing healthcare in the community, food outlet participants acknowledged that the health service may assist in promoting healthy options around the town or food outlet participants who had healthy options available for the health service clientele.

‘*Even if we still had the menus and that put into (the health service) that they can pass on to their clients and things like that*’

Interview participant #1 (Food outlet)

Participants suggested health service endorsement of businesses could also be an effective incentive for food outlet participants to try to make their outlets healthier.

‘*Maybe not here, but elsewhere like in the doctor’s office or something. I know it sounds a bit weird, but you know advertising for sandwich for us there, but—Yeah, outside the business*’

Interview participant #3 (Food outlet)

#### 3.3.4. Characteristics of Desirable Healthy Food Environment Interventions

All participants agreed that there were multiple options for interventions targeting the healthiness of food environments in rural areas, as well as a role that the local rural health service could play in educating food outlet participants. Incentives for food outlet participants and supporting food outlet participants to promote healthy food options using social media to draw the community in were core themes.

‘*I have a big following on social media—Facebook and Instagram. I can’t believe it. The minute I post something, I get busy*’

Interview participant #1 (Food outlet)

Such advertisement was seen as beneficial, whilst many food outlet participants emphasised the need for intervention changes to be easy to implement, such as the use of a sticker or logo to identify a healthy option that is endorsed by the local health service. Some food outlet participants mentioned that physical displays are an ideal target for intervention and could help when advertising healthy options, but to keep the burden on food outlet staff to a minimum.

‘*…we probably wouldn’t wanna go anything too overly taxing for us or the staff, I mean, we’ve got enough to do at the moment as it is, but if it’s just putting a sticker on a label saying this is a healthy option, then it’s a no brainer*’

Interview participant #10 (Food outlet)

Participants suggested that another option could be through the advertisement of healthy options provided by local food outlets, with linkages to the rural tourism market considered by many food outlet participants as an important avenue to explore.

‘*…(the intervention outlets) Could be included in tourism marketing so that when people come here they can look for healthy options*’

Interview participant #2 (Food outlet)

This was further emphasised by the local government, noting the need for convenient healthy food options for tourists travelling through the region.

One participant suggested that incentives for food outlet participants could be a way forward as an intervention but acknowledged the complexity of this.

‘*I think there’s ways that we can incentivise good practice that maybe looks like providing healthy food options, you might get a discount on your rent……I think it would be us (local government) acting alone when an entire industry is built on unhealthy sponsorship of sports.*’

GMB2 Participant #3 (Local Government)

In terms of moving towards a healthier food environment intervention, participants emphasised accounting for the unique social fabric of the rural community context and expressed concern about other food outlets and that an intervention would need to not disadvantage outlets that do not participate.

‘*I call it small town syndrome, but it is working together, networking*’

Interview participant #1 (Food outlet)

Another food outlet participant also emphasised how an intervention must consider the connectedness of rural communities.

‘*It’d have to be pretty careful to not alienate the ones that don’t do it. Yeah. So, that’s probably—I think you got to take that into consideration*’

Interview participant #9 (Food outlet)

There was also a suggestion that food outlet participants would help each other and take a unified approach to improving the healthiness of the food environment as members of the rural community, emphasising the opportunity for impact across food outlet participants.

‘*I think small businesses should help promote other small businesses. My whole thing is to be unique. And I think if we’re talking amongst each other, as much as we could get ideas and gauge support from them, it doesn’t necessarily mean we’re going to run—all of us are not gonna have beef stroganoff on the same day, but we can bounce ideas around and just help promote each other*’

Interview participant #4 (Food outlet)

## 4. Discussion

This study sought to understand the barriers and enablers to promote healthier rural food environments and is the first to explore the perceived role of rural health services in driving healthier food environments from key stakeholders in a rural context in Victoria, Australia. The study also sought to explore desirable intervention strategies applicable to rural communities from the perspectives of food outlet participants, health professionals, the health service, and local government. Despite identifying multiple barriers, participants identified many enablers to promoting healthier food environments, including the potential leadership role of the local rural health service and the strong supportive networks among the food outlet participants within the rural town. 

This is the first study to our knowledge that included health service leaders’ perceptions of the barriers, enablers, and potentially feasible strategies in improving rural food environments and the first to use system science techniques. A recent international review by Vargas et al. that sought to identify which key stakeholders had been involved in co-creating retail interventions found that the most common stakeholders included researchers, corporate representatives or store owners, and governments [34]. The review did not report any studies that detailed the involvement of health service leaders from the rural context in healthy food retail intervention co-creation [34]. The review also only identified 20 eligible studies globally, including 4 Australian studies [34], highlighting the scarcity of literature on understanding ways to improve food environments from key stakeholder perspectives. Further, in contrast to the existing literature around retailer resistance to healthy food retail interventions, this study shows that in some contexts, there may be positivity and willingness among retailers to engage in healthy food retail interventions. 

A recent Australian study by Whelan et al. [35] that evaluated the impact of improving the healthiness of food offered in the on-site café of a rural health service provides evidence of the health-promoting role rural health services can have in their communities, yet empirical data are generally lacking. This is despite health promotion plans outlining role requirements to undertake community-facing action in integrated health promotion plans and the potential for food environment interventions to be a focus of this, harnessing the close context of rural communities [12]. Broadly, rural health services have been identified as key health leaders in their communities [7], and their potential to influence and support local food retail interventions requires further exploration, alongside their government counterparts, who, in this setting, have jurisdictional powers over retailers from a food safety perspective only.

Pre-existing contractual agreements with manufacturers, local government planning policy, community mental health stressors, a preference for convenience, and poor access to healthier foods were viewed as barriers to promoting healthier food environments in the rural context. Although evidence from rural Australia is scarce, the findings reflect the broader international literature on the knowledge of barriers to improving the healthiness of food environments. 

Although the role of rural health services is not well explored, there is more evidence of the role of local governments in healthy food retail [36]. Under the Australian Food Act 1984 [13] and Victoria’s state planning system [37], local governments have no power to regulate the healthiness of food offerings of registered food retailers or those that apply for permits to establish a food retail business [38]. Under the Public Health Act, they are also legislated to have health and wellbeing plans tailored to their local communities [14]. In 2013, the International Network for Food Obesity/NCD Research, Monitoring, and Action Support (INFORMAS) introduced the Government Healthy Food Environment Policy Index (Food-EPI), which recommends that good practices for food retail include policies and practices in place that support the availability of healthy foods in communities and in-store [39]. More specifically, good practices in food retail would see support systems in place to encourage food retailers to promote the in-store availability of healthy foods whilst limiting unhealthy food availability [39]. Aligning with the suggestions raised by food retailers, the Food Policy Index (developed to assess the food and diet-related policies in each jurisdiction in meeting Food EPI’s best-practice recommendations) recommended in 2016 that the Victoria state government implement programs that incentivise or reward (i.e., ‘healthy food’ accreditation schemes, where food retailers receive an award) food retailers improving the healthiness of options [40]. However, the Food Policy Index progress update in 2019 indicated that limited action had been taken to address this [40]. Interventions that support outlets to improve the healthiness of food offerings, which integrate multiple key stakeholders’ perspectives, are integral to increasing the healthiness of food available within communities. It may be that health services and not local governments could be key to driving the implementation of such initiatives in the rural context.

Evidence of possible solutions and the co-creation of interventions that target local restaurants and cafes in rural areas is lacking globally [41]. A review by Alston et al. identified only 20 studies conducted globally on initiatives to improve food environments in rural settings [41]. The review found that effective interventions were characterised by having initiatives that focused on the promotion and awareness of healthy foods and included co-design to generate community ownership and branding [41]. This was also recognised by stakeholders in this study who identified some similar aspects such as using local branding of the intervention and including health service logos that highlight the food outlet’s progress in facilitating healthier environments. Food outlet participants also suggested the use of social media to promote healthy options in-store, incorporating advice from local health service dietitians on menu items, and time-saving strategies such as using stickers to show healthy food options. 

### 4.1. Strengths

A strength of this study was that data were gathered from local government, health service, and local food outlet participants and utilised an in-depth qualitative exploration using both systems dynamics and theoretical perspectives with key informants involved in the rural context. This semi-structured approach allowed interviewers to ask more tailored questions based on responses, which allowed for further in-depth exploration of retailer perspectives [42]. The food outlet participants were also café and restaurant owners, which is novel as the majority of research has focussed on healthy food access in grocery/supermarket settings [41] and extends the understanding of this important out-of-home food source. 

### 4.2. Limitations

The limitations of this research include that the findings are directly applicable only to the rural context and may not be generalisable to all rural communities, as well as only being specific to the café and restaurant retail settings. We also had a relatively small sample of retailers, which may have offered wider perceptions; however, we did reach data saturation as is recommended for qualitative research methodologies. Despite this, the findings may inform and support future action or research in other rural communities that have similar geographical and demographic profiles [43]. Further, not all retailer outlets participated, and it is possible that participants here were more biased toward promoting healthier food environments. Wider community and consumer consultation would have also enriched these data.

## 5. Conclusions

This study shows the complexity of addressing rural food environments and is the first to explore this from the perspective of rural health services, local government, and food outlet stakeholders. Rural food outlet owners perceived the local health service to be a potential knowledge resource that could support and promote food outlets through utilising their unique health leadership position in rural communities to drive change. Key actions to improve rural food environments need to consider the potential role of rural health services, key stakeholder perspectives, and potential interventions palatable to retailers (such as branding and incentives) to assist with community buy-in. Actions to address the healthiness of food environments are needed to make further progress in addressing rural health inequities. 

## Figures and Tables

**Table 1:** Key themes and sub-themes.

Theme	Subtheme
Enablers to healthier food environments	Access to local and appealing healthy food options Promotion of healthier options in stores and on menus Importance of community and social conscience Consumer demand for healthy options and foods that meet dietary requirements
Barriers to healthier food environments	Consumer demand for less healthy options and preference for convenience Contractual agreements with manufacturers that promote unhealthy food supply Local Government Planning policy that does not inhibit the number of unhealthy food outlets Community mental health stressors Poor access to high quality healthy food Lack of knowledge and skills among retailers for understanding healthy food options
Rural health services are a leading broker of knowledge for healthy food environments	Utilising community perception that the health service is the ‘health leader’ in the town Health knowledge brokerage for helping retailers to decipher between dietary requirements and healthy options Advertisement/social media on health service channels to promote businesses who are making efforts to promote healthier food environments Health service endorsement of healthy food businesses Support from dietitians and health promotion officers
Characteristics of desirable healthy food environment interventions	Important to ensure the intervention doesn’t exclude other rural food retail businesses Incentives for businesses who are promoting healthier food environments Assistance with Social media/advertisement of healthy options available Health-service endorsed sticker/logo to identify healthier options Keep the implementation burden low for food outlets Link the outlets who are promoting healthy environments to tourism promotion, to increase business

## Data Availability

We do not have ethics approval to share the data from this study.

## Supplementary Materials

The following supporting information can be downloaded at: https://www.mdpi.com/article/10.3390/nu15234979/s1, Table S1: Themes & Definitions.

## Appendix A. Semi-Structured Interview Guide for Food Outlet Participants

### Appendix A.1. Moderator Verbal Introduction

Thank you so much for making the time for our interview today. Your participation in this research is very important, and we appreciate your time. We have asked you to come here today because we need your help with a research project. I expect the interview will not take longer than 30 min.

Rural Australians have higher rates of preventable disease than their friends living in the cities, and we really want to address this. We are wanting to know more about how rural health services can contribute to improving food environments and preventing chronic disease in our communities.

I am recording this interview today. The interview will be transcribed and de-identified, so your responses will be made anonymous. We will then analyse the interviews into themes and write a research report on this.

Now, if you are happy to, let’s get on with the discussion.

### Appendix A.2. Semi-Structured Interview Guide Discussion Points

1. What affects the food retail environment in <town>? (suppliers, seasons)
2. How will these change as time goes on?
3. If we don’t make any changes, how will this impact the health of <town> residents?
4. How can we promote healthy food options already present in stores?
5. Are there any factors that are connected to each other, or affect each other in some way?
6. What can we (<health service> staff) do to help you as a retailer to improve/advertise the healthy food options?
7. Are there any areas of this map that you think needs attention? (does anything look wrong or need clarification?)
8. Are there large, important issues missing from the map that should be included?
9. If we were to run an intervention to trial some of these changes, what are some challenges stopping you from being involved? For example, customers wants/needs, staffing.
10. If you could be involved in making some of these suggested changes within your business, how long would you like to trial this for? (4 weeks, 12 weeks?)
11. To improve the food environment in <town>, what would be your top priority?

*Prompt: Can you tell us some more about…*
